# A Selective Emotional Decision-Making Bias Elicited by Facial Expressions

**DOI:** 10.1371/journal.pone.0033461

**Published:** 2012-03-15

**Authors:** Nicholas Furl, Shannon Gallagher, Bruno B. Averbeck

**Affiliations:** Unit on Learning and Decision Making, Laboratory of Neuropsychology, National Institute of Mental Health, Bethesda, Maryland, United States of America; French National Centre for Scientific Research, France

## Abstract

Emotional and social information can sway otherwise rational decisions. For example, when participants decide between two faces that are probabilistically rewarded, they make biased choices that favor smiling relative to angry faces. This bias may arise because facial expressions evoke positive and negative emotional responses, which in turn may motivate social approach and avoidance. We tested a wide range of pictures that evoke emotions or convey social information, including animals, words, foods, a variety of scenes, and faces differing in trustworthiness or attractiveness, but we found only facial expressions biased decisions. Our results extend brain imaging and pharmacological findings, which suggest that a brain mechanism supporting social interaction may be involved. Facial expressions appear to exert special influence over this social interaction mechanism, one capable of biasing otherwise rational choices. These results illustrate that only specific types of emotional experiences can best sway our choices.

## Introduction

Visual scenes such as smiling and attractive faces, appetizing foods and beautiful or horrific pictures can evoke strong emotions. People routinely employ such emotional imagery in the media and even during ordinary social interactions to attempt to bias the decisions of others. Although decision making is subject to bias [1], psychology and economics research has classically relied on an assumption that decisions are based on an optimal, unbiased assessment of evidence. This assumption is embodied, for example, by models of instrumental learning. In instrumental learning tasks, an agent learns which choices predict reward or punishment based on feedback from previous choices. Reinforcement learning models [2] can explain instrumental choices by predicting how a rational agent or “ideal observer”, who is not subject to any emotional biases, should evaluate the evidence to make choices that maximize rewards.

Reinforcement learning models are also popular in neuroscience because they describe some of the brain's mechanisms that may control instrumental learning [3]. Reinforcement learning models compute reward prediction errors from choice feedback and these errors can accurately predict neural responses in the mesostriatal dopamine “reward system” [4]–[6] and its targets including the ventral striatum [6], [7]. Thus, these reward-related areas appear to contribute to feedback-based learning, and evidence suggests they implement a mechanism akin to that of reinforcement learning models.

While the reward system may contribute to rational decisions about monetary rewards, different brain mechanisms may contribute when decisions are biased by emotional or social information. Averbeck & Duchaine [8] devised an instrumental learning task that required choosing between images of a smiling or angry expression, where the two images were respectively associated with a 0.60 and a 0.40 chance of winning £0.10. Although participants were instructed to maximize their winnings, they were biased to choose the smiling face over the angry face, given equivalent feedback. This was in contrast to a reinforcement learning model which showed no bias. Evans, Fleming, Dolan & Averbeck [9] examined the instrumental learning task using functional magnetic resonance imaging (fMRI). As predicted by reinforcement learning models, reward prediction errors to monetary feedback were indeed associated with responses in the ventral striatum. However, the expression bias was correlated instead with responses in temporoparietal junction, a component of the putative “mentalizing network”, responsible for understanding the thoughts and intentions of others [10]–[12]. The possibility that social interaction mechanisms mediate the bias is in line with findings that angry facial expressions facilitate social avoidance-related behavior [13]. Moreover, in a further study, the size of the bias was modulated by oxytocin [14], a neuropeptide known to increase social approach behavior and enhance face expression recognition [15]–[19].

This brain imaging and pharmacological data suggest involvement of a mechanism related to social interaction. Nevertheless, it is possible that the biases are due to their positive and negative emotional valence and not the specifically social component. In this case, any stimulus pair which similarly differs in emotional valence will recruit the same mechanisms and produce a bias. A second untested possibility is that socially-relevant dimensions other than emotional expressions, including attractiveness and trustworthiness, may also bias choices in decision behavior.

While it is possible that the bias is restricted to certain types of stimulus information, it may also be restricted to certain kinds of decisions. To date, this bias has been observed only for learning based on monetary rewards. However, learning based on monetary rewards and learning based on monetary losses (punishments) involve neural mechanisms that differ in their dependence on dopamine [6] and are partially distinct anatomically [20]–[22]. Facial expressions therefore might modulate only one of these mechanisms.

We investigated the specificity with which emotional and social information can bias otherwise rational instrumental learning. In the first experiment, we compared the influence of facial expressions with that of emotionally-valenced images of animals and food items. The second experiment employed a wider range of non-face image pairs, including more animals and food items and various emotional scenes and words. For the third experiment, we measured the bias for face pairs differing in trustworthiness or attractiveness. For the fourth experiment, we obtained emotional valence ratings of the images used in the preceding experiments for correlations with bias size. Finally, for the fifth experiment, we used facial expressions to measure the bias when learning was based on monetary rewards or losses.

## Methods

### Ethics statement

We recruited participants from the National Institutes of Health and surrounding community. Written consent was obtained from all participants following a protocol approved by the National Institutes of Health Institutional Review Board.

### Participants

No participants had any psychiatric or neurological disorder, as verified by a staff physician. The five experiments derived from three samples of participants. The first sample participated only in Experiment 1. After one male participant was excluded due to equipment error, the remaining 15 males and 6 females had an average age of 28.3 years (range 22–49 years). The second sample participated in Experiment 2 (5 males; 16 females; average age 24.8, range 22–30). Experiments 3, 4 and 5 consisted of data from a third sample of 16 participants (8 males, average age 28.5, range 21–54). In this group, the ratings task (Experiment 4) was always completed first to ensure all rated images were equally unfamiliar to all the participants. Participants then underwent Experiments 3 and 5, with their order counterbalanced across participants.

### Instrumental learning task

In each experiment, we implemented instrumental learning tasks in several “conditions”. Each condition was a separate run of the experiment and consisted of four blocks of 26 choice trials. In each block, two images were respectively associated with a 0.60 and a 0.40 chance of a $0.10 reward (although see loss blocks in Experiment 5). Participants were instructed before each new block that the images were assigned new probabilities and to make decisions to maximize their money. Each image pair consisted of a ‘positive’ and a ‘negative’ image. The positive and negative images were presented side-by-side on each trial, such that each appeared on the right side of the screen for a pseudorandomly chosen 50% of the trials in each block. Participants chose the right or left image by keypress. When participants were rewarded, a screen appeared stating ‘you win 10 cents!!’ and the current winnings. If there was no reward given in the trial, a screen appeared stating ‘you lose.’ Two emotionally-valenced image pairs were presented in each condition, one pair seen in the first and third blocks and the other pair in the second and fourth blocks. For faces, the two image pairs were always different identities. For the two blocks within a condition that corresponded to a given image pair, the positive image was more likely to be rewarded in one block, and the negative image more likely to be rewarded in the other. The order of probability assignments to the positive vs. the negative image was randomized across subjects.

### Data analysis

All analyses were carried out in MATLAB (The MathWorks, Natick, MA). For the ideal observer model, we used a simplified variant of the reinforcement learning model previously described in detail [8], [9], [14]. For the analyses reported here, the simplified model provides equivalent predictions. The model used in this study prescribed the optimal choice between the two images available on each trial, by selecting the image with the most “evidence”, defined as the proportion of rewards resulting from preceding choices of that image in the block. For example, if, on a given trial, each face had been chosen twice, and the angry face had won $0.10 both times (2 wins/2 choices = 100% evidence), while the smiling face had won once and lost once (1 win/2 choices = 50% evidence), then the evidence would favor the angry face. At the beginning of a block, the evidence for each image was assigned a value of 0.50 (chance) until it was chosen, when its value was then determined by the choice outcomes, as described above. On some trials, participants agreed with the ideal observer model and correctly chose the optimal image based on the evidence, while on other trials, participants made choices that disagreed with the evidence. The relationship between participants' and model's choices (evidence-based), then, could be represented by a 2×2 contingency table, which tabulated each time a participant's choice agreed or disagreed with the evidence. In this table, if the evidence favoring each image on a trial was equal, we tabulated a 0.5 for both possible model choices, denoting equal agreement for both outcomes. For all conditions reported below, participants agreed with the model more often than predicted by chance (*P*<0.05, Bonferroni corrected). For cases when participants disagreed with the model, we computed the conditional probability that each participant would choose the positive image given that the evidence in fact favored the negative image – “positivity errors”. Similarly, “negativity errors” are measured as the conditional probability that each participant would choose the negatively-valenced image given that the evidence favored the positive image. When negativity errors are subtracted from positivity errors, the resulting quantity measures the bias. That is, it indicates how often participants irrationally ignored previous evidence and chose the positive image, compared to how often they irrationally chose the negative image.

All ANOVAs used at least the following two fixed effects factors. The factor Error Type contrasted positivity and negativity errors (the bias) while the factor Condition contrasted the different types of image pairs considered in each experiment. The nature of the conditions varied depending on the experiment. We were particularly interested in the Error Type×Condition interaction, which tests whether the difference between error types (i.e., the bias) differs among the conditions. Further, we included Participant as a nuisance factor and also Rewarded Stimulus, which contrasted blocks where the positive image had a higher probability of reward against blocks in which the negative image had a higher probability of reward. In a separate analysis, we performed additional ANOVAs, including a gender term for the participants; there were no significant main effects or interactions including gender. We employed similar ANOVAs to test post-hoc whether pairs of conditions within an experiment differed in bias, while also controlling for nuisance variability in Rewarded Stimulus and Participant. We report Bonferroni-corrected *P*-values for these post-hoc *F*-tests. We lastly performed Bonferroni-corrected *t*-tests to test if any condition's bias differed from zero.

## Results

### Human and animal facial expressions bias decisions more than foods

**Figure 1:**
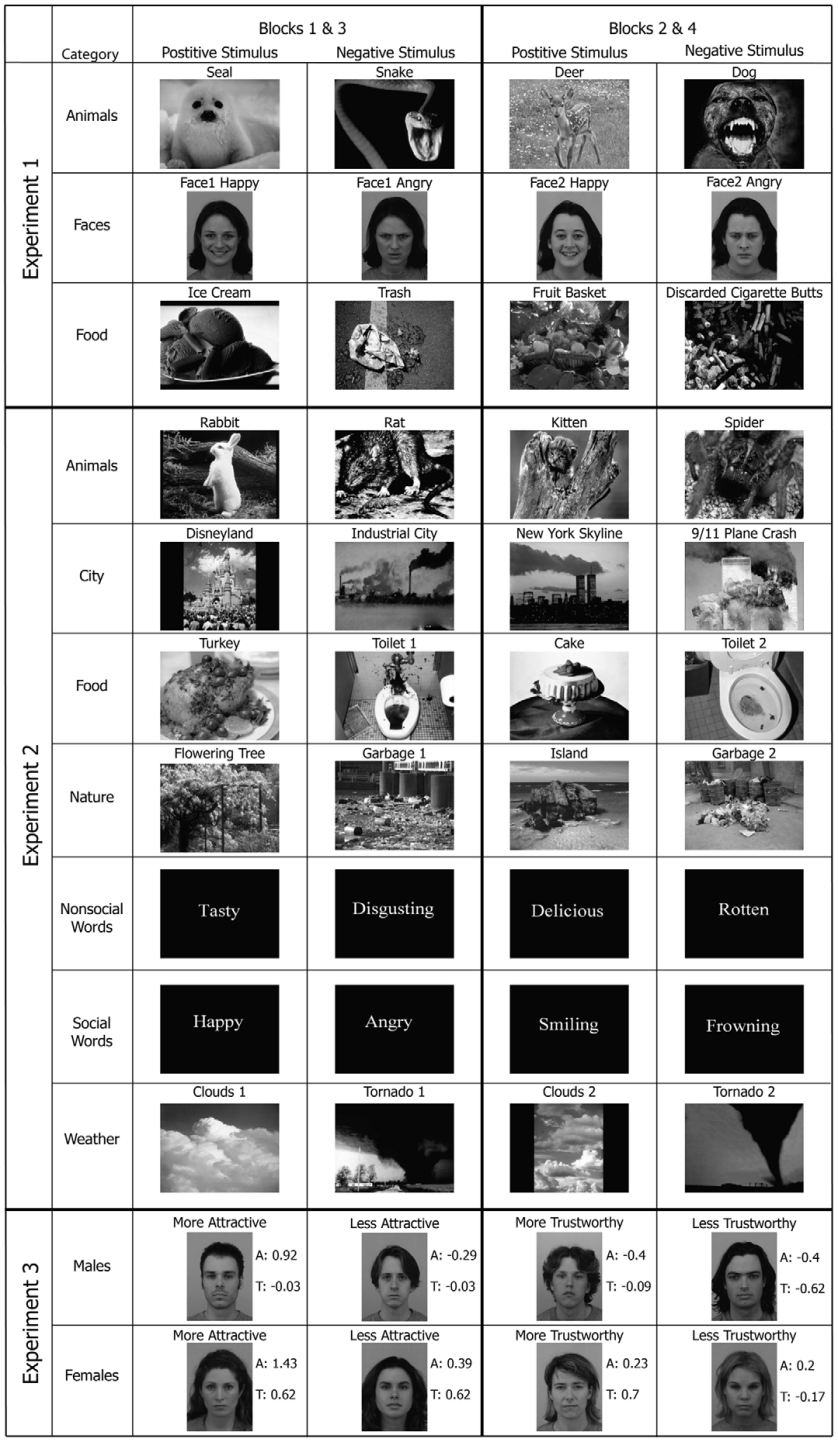
Emotionally-valenced image pairs used as stimuli in Experiments 1–3.

**Figure 2:**
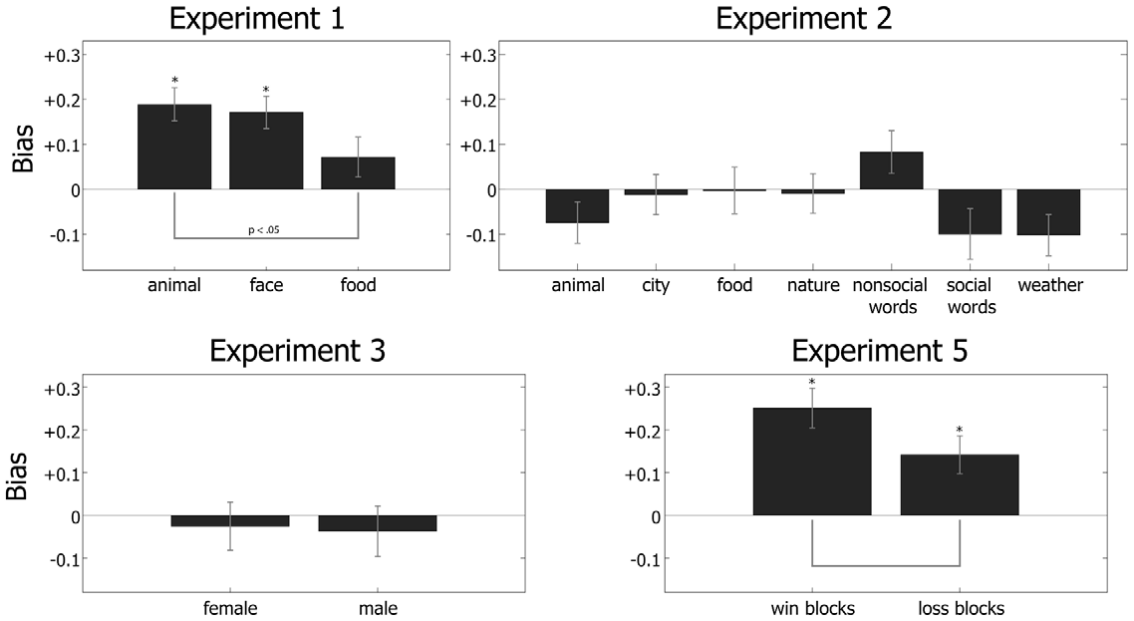
Mean and standard errors across participants of the biases for each condition in Experiments 1, 2, 3 and 5.

Smiling and angry expressions may respectively evoke positive and negative emotional responses in participants and this difference in emotional valence may be sufficient to bias decisions. We therefore compared pictures of facial expressions with various types of images that can evoke positive and negative emotional responses and tested whether these valenced image pairs would give rise to comparable-sized decision biases. We investigated images of animals which were cute [23] or threatening and images of food items, which were appetizing or repulsive (Fig. 1), taken from the International Affective Picture System database (IAPS) [24]. We compared these with smiling and angry facial expressions using two female identities taken from the Karolinska Directed Emotional Faces (KDEF) database [25]. The three conditions were counterbalanced in a Latin square across participants. The three conditions differed in the size of their bias (Fig. 2), as shown by a significant Error Type×Condition interaction, *F*(2,332) = 3.55, *P* = 0.0299. Planned comparisons showed that biases towards the positive affective image were greater than zero for both faces, *t*(83) = 4.75, *P*<0.05, and animals, *t*(83) = 5.13, *P*<0.05, but not for food items, *t*(83) = 1.60, *P*>0.05. Animal images also showed a larger bias than food items, *t*(83) = 2.55, *P*<0.05. The absence of bias for food items suggests that not all forms of emotional valence can bias decisions, but that some types of emotional information may be more effective than others. Cute and threatening pictures of animals, like facial expressions, may evoke emotions that motivate social approach and avoidance [23], whereas appetizing and disgusting food items do not. Our results, however, are also possible if facial expressions more specifically bias decisions. The animals in Experiment 1 not only have visible faces, but the negative animal pictures (snake and dog) manifest obviously threatening facial expressions. We will investigate animal pictures without recognizable emotional expressions in Experiment 2.

### Emotional images without facial expressions do not bias decisions

In Experiment 2, we more extensively explored different types of positive and negative image pairs. As in Experiment 1, we were interested in determining whether emotional valence is sufficient to bias decisions. We also investigated whether pairs of stimuli with social relevance would give rise to larger decision biases, as this might explain the bias to animal images observed in Experiment 1. In Experiment 2, we tested seven categories of non-face stimuli (Fig. 1): animals, food items (as in Experiment 1), city scenes, nature scenes, non-social words, social words, and weather scenes. With the exception of the words, these stimuli were all drawn from the IAPS database. Unlike Experiment 1, we chose animal image pairs without recognizable facial expressions. The seven image categories were presented as separate conditions, with order counterbalanced in a Latin square across participants. The variation in bias size among the category types (Fig. 2) was not sufficient to yield a Category×Error Type interaction *P*>0.118. Moreover, no category showed a bias significantly different from zero. When differences in bias among all pairs of categories were tested, we found no significant effects (*P*>0.107 corrected). Thus, like Experiment 1, we found no evidence that emotional valence is sufficient to bias decisions. Even positive and negative stimuli which were socially-relevant failed to show bias, including words that communicated social content. Even though positive and negative animal pictures might motivate social approach and avoidance, they bias decisions only when facial expressions are present (Experiment 1). Experiment 3 uses facial stimuli to more directly investigate the biasing capability of image pairs differing in potential social approach and avoidance.

### Facial trustworthiness and attractiveness do not bias decisions

We tested whether social approach/avoidance information in faces, other than emotional expressions, can bias decisions. We chose human faces with neutral expressions that differed in either perceived attractiveness or trustworthiness (Fig. 1) and tested whether differences in these social attributes showed the same influence on decision making as facial expressions. We used previously published attractiveness and trustworthiness ratings of pictures in the KDEF database [26] to choose faces based on the largest difference in one characteristic (attractiveness or trustworthiness) while holding the other one as constant as possible. There were four blocks in Experiment 3. In blocks 1 and 3, the faces viewed had similar trustworthiness ratings but differed in attractiveness. In blocks 2 and 4, the faces had similar attractiveness ratings but differed in trustworthiness. Neither faces that differed in attractiveness nor those that differed in trustworthiness showed any bias (Fig. 2), as there was no significant Error Type×Condition interaction, nor were any post-hoc tests significant (*P*>0.056). Thus, we have no evidence to claim that any social attribute other than facial expressions can influence instrumental learning.

### Emotional valence does not correlate with the bias

**Figure 3:**
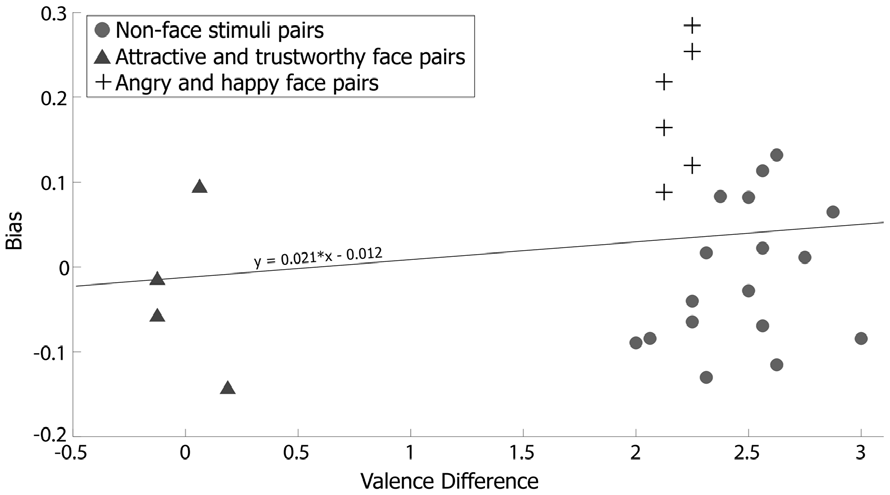
Results from Experiment 4. The scatter plot shows relationship between bias size and the valence difference for every image pair used in Experiments 1–3 including non-face images (grey circles), faces differing in trustworthiness or attractiveness (black triangles) and facial expressions (black crosses).

One possibility that might explain the absence of results in Experiments 1, 2 and 3 is that the image pairs chosen did not evoke as strong or differentiated emotional responses in our participants as facial expressions. If emotional valence generally biases decisions, then facial expressions may appear to selectively bias decisions because their emotional valence is more salient than for other pictures. We tested in Experiment 4 whether the difference in emotional valence between each image pair related to the size of the bias. We presented to participants every picture used throughout Experiments 1–3 and 5 in a pseudorandom order and they made a self-paced rating of each on a scale from one to five where 1 = extremely negative; 2 = negative; 3 = neutral; 4 = positive; 5 = extremely positive. We subtracted the mean rating for each negative image from that of the corresponding positive image for each image pair used in the instrumental learning task to derive differences in valence. We then correlated the valence difference for each image pair in Experiments 1–3 and 5 with the corresponding bias. We found no significant correlation (*ρ* = 0.160, *P* = 0.425)(Fig. 3). Non-face stimulus pairs (grey circles) were nearly all rated with equivalent or higher valence than the face expression pairs (black crosses), yet the biases associated with non-face image pairs were distributed around zero. This provides further evidence that emotional valence in general is not sufficient to induce instrumental learning biases.

### Decisions are biased for either rewarding or punishing outcomes

Experiments 1–4 suggest that the bias was mediated by a mechanism for which face expressions are the most effective inputs of those that we examined. We also investigated the outputs of this mechanism by exploring the types of instrumental learning that can be affected by facial expressions. Specifically, we tested whether the facial expression bias was specific for learning from monetary reward, or whether the bias was present when participants learned from punishment as well. We compared the size of the bias in conditions where the images differed in potential monetary wins (a replication of Experiment 1) versus potential monetary losses. The face pairs used were identical to those used in the faces condition of Experiment 1 (Fig. 1). Participants began Experiment 5 with a pot of $6.00, and then underwent counterbalanced win and loss conditions (four blocks in each). The running balance for the total of eight blocks was recorded continuously. In the win blocks, the task was identical to Experiment 1. In the ‘loss’ blocks, one image had a 60% chance of loss, while the other had a 40% chance of loss. Participants were again instructed to attempt to maximize their money. If the participant lost money, a screen would appear stating ‘you lose 10 cents!’ and displaying the current monetary sum. Otherwise, a screen would appear stating ‘no loss!’ Although the bias in the wins condition was numerically larger than that of the losses condition (Fig. 2), the Error Type×Condition interaction did not reach significance, *F*(1, 225) = 3.57, *P* = 0.21. Moreover, decisions were significantly biased for both win *t*(63) = 5.55, *P*<0.05 and loss *t*(63) = 3.21, *P*<0.05 conditions. Thus, we have insufficient evidence to conclude that the expression bias is selective for reward-related decisions versus punishment-related decisions.

## Discussion

We investigated the specificity of emotional/social biases on instrumental learning. We replicated the finding that participants' reward-related decisions favored smiling over angry expressions, when these choices were not supported by evidence from previous choices. This bias occurred only for expressions and not for the other types of emotionally-valenced image pairs that we investigated, even when they were socially-relevant. In sum, the biasing influence of emotions on decision-making is restricted and facial expressions appear to have a special importance.

We were interested in whether the bias previously observed for facial expressions [8], [9], [14] also related more generally to social relevance or emotional valence. We therefore examined a variety of emotionally-valenced image pairs that had social or non-social content but none produced biases nor did the perceived emotional valence of the pairs relate to the bias size. Indeed, facial expressions, which showed a bias, were rated as less valenced than most non-face stimuli, which did not show any bias. We found that animal pictures, which might induce social approach or aversion in our participants [23], gave rise to a bias only when threatening facial expressions and ‘inviting’ faces were visible. We also tested words expressing social content comparable to that of facial expressions (“happy”, “angry”) but these did not bias decision making either. Lastly, faces differing in social attributes including attractiveness or trustworthiness did not give rise to any bias. Thus we may eliminate emotional valence and general social-relevance as sufficient to bring about a bias on decision making.

We did not directly observe the brain mechanisms mediating these biasing influences on instrumental learning. Nevertheless, our behavioral methods allow us to re-evaluate existing findings showing that neural mechanisms contributing to social approach/avoidance may mediate the bias. Particularly, the facial expression bias is associated with mentalizing networks, responsible for inferring the thoughts and intentions of others [9] and also oxytocin, an agent known to promote prosocial behavior [14]. Our results qualify these findings, as the biasing influences of these neural mechanisms may not be deployed by social information generally, but instead by a more restricted class of stimuli, including facial expressions.

Although we can clearly claim that the bias is highly selective to some visual stimuli, conclusive claims about selectivity are notoriously complicated by the fact that all stimuli cannot possibly be compared. Nevertheless, the selective findings we show here suggest several new lines of research which can further test which stimuli induce biases and which do not. First, we examined only two social dimensions of the face: attractiveness and trustworthiness. These social dimensions and many others might be investigated more thoroughly by more systematically manipulating the amount of information related to these dimensions in the stimuli. Indeed, artificial facial stimuli can be constructed and tested, which can span the face-space of social dimensions [26]. Second, arousal [27] is also an important dimension in emotion processing not yet investigated. It is possible that stimuli known to be highly arousing, such as erotica, violent animal or human attacks and mutilation, might also lead to decision-making biases via their activation of appetitive and defensive motivational systems [28]. Furthermore, although food-related stimuli did not produce a bias in our study, the arousal produced by these stimuli might be heightened and the bias thereby affected using the physiological and mental states (hunger, thirst) of participants, as well as their personal preferences (e.g. dietary). A third important possibility is that expressions produced a bias because they are bodily actions. Indeed, the general class of emotional bodily actions, including non-face actions, may induce biases. There is a considerable literature examining bodily expressions of emotion [28]–[30]. These stimuli, as well as those depicting acts of violence or erotica (such as those in the IAPS stimulus set) might also predict biases, if the general category of emotional bodily actions is sufficient to bias decisions.

Our results also have practical applications for understanding how people respond to persuasive information. Numerous social contexts, including commercial advertising, routinely employ images of expressive faces as well as many of the types of emotional information that we investigated here. These presentations are intended to convince others that a decision (such as the one advertised) will lead to a rewarding outcome or will cause an adverse outcome to be avoided. Our results provide evidence that emotional expressions are indeed effective influences on actions and expectations of rewards. While attractive faces, descriptive language (social or nonsocial words) or stirring or appetizing images might attract attention and induce potent emotional or appetitive responses, our data suggest this content may be less effective in altering choices. Moreover, facial expressions led participants to ignore evidence about potential monetary gains and, to some extent, behave irrationally. As irrational actions may be undesirable, it is important to examine more closely the factors that can attenuate emotional biases so that people can make more reasonable and rewarding decisions.

In conclusion, emotional information gave rise to irrational decisions, where participants expected monetary rewards following smiling expressions compared to angry expressions, when past evidence did not support this expectation. Across a surprisingly wide variety of social and otherwise emotional stimuli, only facial expressions (human and animal) gave rise to this decision-making bias. Actions of other people and animals which convey emotion may exert a special influence on how people make decisions. These results, and the study of emotional deviations from rational decision-making in general, are important for understanding how emotions can influence our actions and choices.
